# Myxoma of the Ovary

**DOI:** 10.4274/balkanmedj.2017.0842

**Published:** 2018-01-20

**Authors:** Recep Bedir, Rukiye Yılmaz, Ahmet Salih Calapoğlu

**Affiliations:** 1 Department of Pathology, Recep Tayyip Erdoğan University School of Medicine, Rize, Turkey; 2 Department of Pediatric Surgery, Recep Tayyip Erdoğan University School of Medicine, Rize, Turkey

Myxomas are benign tumors that generally arise from the heart, soft tissues, muscle, skin and bone (1). Herein we present a myxoma case found on the ovary.

A 12-year-old girl was under follow-up for menstrual irregularities for 1 year and pelvic ultrasonography revealed bilateral para-ovarian simple semisolid lesions. Pelvic magnetic resonance imaging was performed, and a cystic lesion (65×4 cm) in the right adnexal region that was hyperintense on T2-AG and hypointense on T1-AG was observed (Figure 1a). Ovarian tumor markers and routine laboratory evaluations were all within normal limits. The right adnexal para-ovarian cystic mass was completely excised. Macroscopically cutting the surface of a cystic tissue sample totally 6×4.5×3 cm dimensions was bloody, and a solid myxoid area of 3×2.5 cm was observed on one side (Figure 1b). Microscopically, there was a tumor consisting of stellate or spindle-shaped tumor cells in a large myxoid matrix (Figure 1c). There was not any increase in cellularity, nuclear polymorphism, mitosis or necrosis determined in the tumor (Figure 1d). Histochemically, positive staining with Alcian blue was observed in myxomatous stroma (Figure 1e). Immunohistochemical evaluation revealed diffuse positive staining with smooth muscle actin and vimentin, and negative staining with desmin, inhibin, cytokeratin and S-100 protein in the tumor (Figure 1f). Based on these findings, the patient was diagnosed with ovarian myxoma.

Written informed consent was obtained from the patient before the aforementioned work was carried out.

Ovarian myxoma is quite a rare condition that is a rare entity (2). Histologically, these tumors are produced from the cells in a random sequence in a myxoid matrix without an infiltrative growth pattern, mitotic activity, cytological atypia or necrosis (3,4).

The differential diagnosis of ovarian myxomas with other ovarian myxoid lesions should be performed. The differential diagnosis includes angio-myxomas, fibromas with widespread myxoid degeneration, and primary ovarian stromal lesions containing high amounts of myxoid matrix, such as massive edema, or non-ovarian stromal lesions that are generally maligned (2,5). In massive edema of the ovaries, the normal residual ovarian stromal and intercellular structures together with the mucin negativity in connective tissue are observed around the edematous tissue. Fibromas with myxoid degeneration comprise normal fibrous tissue in some areas. Aggressive angiomyxoma may commonly mimic ovarian myxoma both with immunohistochemical and ultrastructural findings. In aggressive angio-myxomas, there are infiltrative borders with finger-like invasions through the surrounding fat tissue together with a definitive thick-walled vascular pattern. In angio-myxomas, the intercellular matrix shows weak staining with Alcian blue while a dense positive staining is observed in ovarian myxomas due to the presence of hyaluronic acid (2,4).

Although ovarian myxomas are benign, their behavior is uncertain and recurrences may be seen (3). In the literature, this condition was explained by the difficulty of total excision of viscous material that may cause recurrences. For that reason, total excision of myxomatous ovarian tumors together with the adnexal structures was advised. Increases in mitotic activity, cellularity or nuclear atypia in tumors are the predictors that aid in an estimation of recurrences (2).

In conclusion, ovarian myxomas are rare, and they should be differentiated from other lesions of the ovaries with myxoid alterations. The preoperative diagnosis is difficult, and the exact diagnosis must be performed with the histopathological investigations. We believe that this tumor should be differentiated carefully from other benign and malign myxoid lesions of the ovaries by the aid of immunohistochemical evaluation.

## Figures and Tables

**Figure 1:**
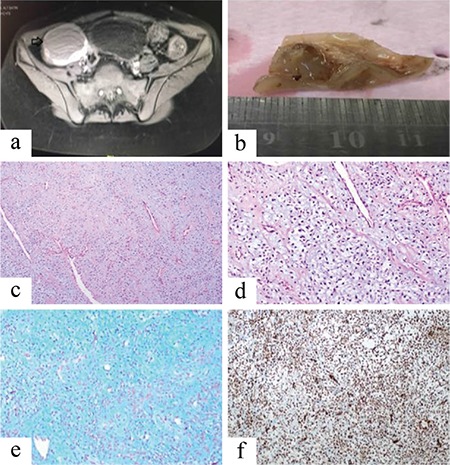
MRI imaging observed cystic lesion on right adnexal region (black arrow) (a), Macroscopically showing multiloculated cystic and solid tumor containing gelatinous material (b), Microscopically showing dispersed oval to spindle-shaped cells with elongated nuclei in a myxomatous stroma (hematoxalin and eosin stain, x100, x200, respectively) (c, d), Alcian blue positivity in myxomatous stroma (x200) (e), Vimentin positivity in tumor cells (x200) (f).
